# Assessment of Spectral Computed Tomography Image Quality and Detection of Lesions in the Liver Based on Image Reconstruction Algorithms and Virtual Tube Voltage

**DOI:** 10.3390/diagnostics15081043

**Published:** 2025-04-19

**Authors:** Areej Hamami, Mohammad Aljamal, Nora Almuqbil, Mohammad Al-Harbi, Zuhal Y. Hamd

**Affiliations:** 1 Department of Medical Imaging, Faculty of Allied Medical Sciences, Arab American University, 13 Zababdeh, Jenin P.O. Box 240, Palestine; areej.hamami98@aaup.edu; 2 Department of Radiological Sciences, College of Health and Rehabilitation Sciences, Princess Nourah bint Abdulrahman University, P.O. Box 84428, Riyadh 11671, Saudi Arabia; noaalmuqbil@pnu.edu.sa (N.A.); zyhamd@pnu.edu.sa (Z.Y.H.); 3 Medical Imaging Department, King Abdullah bin Abdulaziz University Hospital, P.O. Box 47330, Riyadh 11552, Saudi Arabia; moalharbi@kaauh.edu.sa

**Keywords:** liver diseases, spectral detector CT, multidetector CT, reconstruction algorithms, virtual tube voltage

## Abstract

**Background**: Spectral detector computed tomography (SDCT) has demonstrated superior diagnostic performance and image quality in liver disease assessment compared with traditional CT. Selecting the right reconstruction algorithm and tube voltage is essential to avoid increased noise and diagnostic errors. **Objectives**: This study evaluated improvements in image quality achieved using various virtual tube voltages and reconstruction algorithms for diagnosing common liver diseases with spectral CT. **Methods**: This retrospective study involved forty-seven patients who underwent spectral CT scans for liver conditions, including fatty liver, hemangiomas, and metastatic lesions. The assessment utilized signal-to-noise ratio (SNR) and contrast-to-noise ratio (CNR), with images reconstructed using various algorithms (IMR, iDose) at different levels and virtual tube voltages. Three experienced radiologists analyzed the reconstructed images to identify the best reconstruction methods and tube voltage combinations for diagnosing these liver pathologies. **Results**: The signal-to-noise ratio (SNR) was highest for spectral CT images using the IMR3 algorithm in metastatic, hemangioma, and fatty liver cases. A strong positive correlation was found between IMR3 at 120 keV and 70 keV (*p*-value = 0.000). In contrast, iDOSE2 at 120 keV and 70 keV showed a low correlation of 0.291 (*p*-value = 0.045). Evaluators noted that IMR1 at 70 keV provided the best visibility for liver lesions (mean = 3.58), while IMR3 at 120 keV had the lowest image quality (mean = 2.65). **Conclusions**: Improvements in image quality were noted with SDCT, especially in SNR values for liver tissues at low radiation doses and a specific IMR level. The IMR1 algorithm reduced noise, enhancing the visibility of liver lesions for better diagnosis.

## 1. Introduction

Treatment plans and patient management are significantly impacted by liver lesions. Therefore, it is now crucial to precisely evaluate important liver disease parameters using noninvasive techniques like computed tomography (CT) scans and magnetic resonance imaging (MRI) [1]. MRI has certain limitations, even though it is the gold standard for identifying liver diseases. Patients who have metallic implants cannot have MRI scans, and the examination process can take a long time. Noninvasive technologies are therefore desperately needed in a variety of clinical research fields and therapeutic contexts to support early disease detection and longitudinal disease tracking during treatment. Compared with traditional imaging techniques, spectral detector computed tomography (SDCT) imaging has shown superior sensitivity for a variety of diseases, achieving a sensitivity of 77% and a specificity of 95% [2].

SDCT technology offers significant advantages over multi-detector CT (MDCT) by providing comprehensive spectral information to all patients without the need for additional radiation exposure or pre-selective protocol decisions. Additionally, SDCT features full temporal and spatial alignment, complete field-of-view imaging, and minimal rotation times of 0.27 s, all of which enhance patient safety and diagnostic accuracy. Utilizing spectral detectors instead of conventional multidetector CT presents opportunities to minimize radiation exposure, primarily by reducing the need for follow-up imaging or unnecessary repeat studies, as well as potentially omitting specific phases from multiphase research. Similar to other dual-energy computed tomography (DECT) technologies, SDCT can reduce the contrast burden while capitalizing on iodine’s heightened attenuation properties at lower energy levels [3]. Also, SDCT has been found to enhance contrast-to-noise ratio (CNR) and subjective image quality in low keV images for the evaluation of liver diseases such as cysts and hypovascular metastases, improving the detection of hypovascular liver lesions [4]. It can diagnose diseases based on virtual kV without the need to change the dose, in contrast to available devices that must be imaged several times to obtain the best diagnosis. Previous studies have examined CNR in the superior mesenteric artery [5,6] and hepatic blood vessels [7,8]. The highest CNR for abdominal angiograph imaging was observed in 40 keV monoenergetic images [9]. On a typical CT scan, cholesterol-containing gallstones may seem isoattenuating to the surrounding bile. Studies have shown that virtual monochromatic imaging (VMI) using SDCT can increase the visibility of these stones at both low and high energies, with remarkable improvements [10], in addition to enabling the diagnosis of necrotic hepatocellular carcinoma (nHCC) and hepatic abscess (HA) [11]. In cirrhotic patients, dual-energy CT enables the prediction of liver-related diseases [12,13] and the staging of liver fibrosis [14,15]. It can be used to differentiate between abscesses in the liver [16], evaluate fatty liver [17], measure the quantification of iodine in liver lesions [18], and assess early-stage metastasis [19].

From another perspective, many attempts have been made to reduce the radiation dose without compromising image quality, by using iterative reconstruction (IR) techniques instead of traditional filtered back projection (FBP) [20]. FBP is a quick and easy solution that can be obtained from one pass over the acquired data; nevertheless, during acquisition, quantum and electronic noise affect the projection data used in the FBP algorithm. This occasionally magnifies the noise into images, resulting in streaks and other artifacts. As a result, noise and poor image quality are frequently produced when dose reduction is achieved using FBP reconstruction [21]. To overcome these restrictions, many IR techniques have been developed, including a process known as iterative reconstruction (iDose4) that produces lower image noise compared with the traditional filtered-back-projection (FBP) method [22]. Iterative model reconstruction (IMR) produces a lesser amount of image noise. For diagnosis purposes, the generated IMR image may be utilized in place of traditional FBP images. The goal of IMR is to lower the dosage needed for diagnostic imaging using CT. The therapeutic task, the size of the patient’s body, the location of the anatomy, and the clinical setting all influence image quality and the possibility of dose reduction [23]. Therefore, IMR and iDose algorithms could also play an important role in imaging liver disease by reducing the patient’s dose and improving image quality for diagnosis [24]. Some studies have been conducted to assess the effectiveness of SDCT in the diagnosis of several pathologies such as pancreatic diseases, using conventional polyenergetic reconstructions (polyE) and virtual tube voltage (monoE) [25], while other authors have focused on assessing the incremental value of spectral CT data over conventional CT for fully automated localization and diagnosis of liver lesions. Utilizing a spectral dual-layer CT scanner based on virtual monochromatic imaging (VMI) [26], Kulkarni et al. [24] compared iterative model reconstruction (IMR) with low-dose and hybrid iterative CT scanning techniques for evaluating liver diseases like cirrhosis and hepatocellular carcinoma. To the authors’ knowledge, no studies have been conducted to evaluate the effectiveness of combining different levels of iterative model reconstruction (IMR) and iDose with varying virtual tube voltages for diagnosing liver lesions using standard-dose computed tomography (SDCT). Therefore, further research is necessary to confirm the clinical significance of combining IMR and iDose with virtual tube voltage, to evaluate their effectiveness in detecting the most common liver lesions via SDCT imaging.

## 2. Materials and Methods

This retrospective study was conducted with selected patients, with data collected between May and August 2023. The patients in this study had previously undergone imaging using the Spectral CT 7500 (Philips, Best, Netherlands), the only Spectral CT scanner available in the region. Each patient had been diagnosed with one of the following liver conditions: metastatic lesions, hemangiomas, or fatty liver. These conditions are among the most prevalent in this patient population. This study obtained ethical approval from the local institutional review committees (IRB number: R-2023/A/119/N). The distribution and demographic characteristics of the participants in the cases are shown in Table 1.

**Figure 1:**
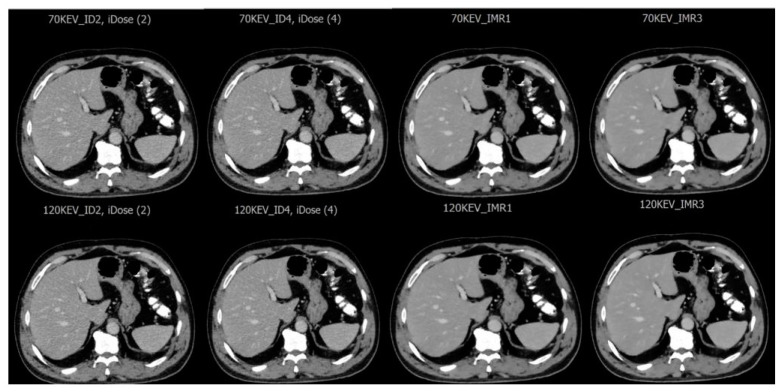
Eight combinations were assessed based on 70 keV and 120 keV and different levels of iDose and IMR reconstruction algorithms.

The study involved 47 patients out of 92 available in the Picture Archiving and Communication System (Stradus v 1, Best, Netherlands). who met the inclusion criteria. The inclusion criteria were as follows. To be eligible for an enhanced CT scan, patients must have already undergone a standard enhanced CT scan and be at least 18 years old. Additionally, there had to be comprehensive documented clinical data available for each patient with one of the selected diseases (liver metastasis, liver hemangioma, or fatty liver). Exclusion criteria included patients younger than 18 years, emergency patients, and those with unconfirmed liver disease, as well as those with incomplete data forms. Each image was reconstructed at two levels: level 2 and level 4 for iDose and level 1 and level 3 for IMAR. To evaluate the impact of tube voltage on the assessment of various pathologies, two tube voltages were used in the virtual CT system: low tube voltage at 70 keV and high tube voltage at 120 keV. Consequently, eight combinations were created for each patient to examine how the reconstruction algorithm and tube voltage affected the diagnostic capability for liver disease (Table 2, Figure 1).

Three experienced radiologists, each with a minimum of eight years of expertise, meticulously analyzed eight combinations of reconstructed CT images. Each radiologist carefully examined a total of 376 scans from 47 patients. High-quality, high-resolution monitors were used during this comprehensive evaluation process to ensure accurate assessments. The analysis primarily focused on the axial reconstructed images within a clinical setting. Following predefined guidelines, the radiologists concentrated on evaluating the sharpness, contrast, and characterization of lesions in the scans.

These radiologists employed a systematic approach by following established protocols to ensure consistency and precision in their evaluations. Their focus on sharpness, contrast, and lesion characterization was essential for making accurate diagnoses of liver diseases. Additionally, to standardize the evaluation process and maintain uniformity in scoring, a 5-point Likert scale was used to quantitatively assess the overall image quality for diagnosing various liver conditions. Scoring was as follows: 1, insufficient image quality; 2, poor image quality; 3, moderate image quality; 4, good image quality; 5, excellent image quality. This scoring system offered a structured framework for the radiologists to consistently and effectively evaluate the images based on their perceived quality and diagnostic value. By employing this methodical approach, the team of radiologists aimed to enhance the accuracy and reliability of their assessments, ultimately benefiting patient care and treatment outcomes.

### Image Quality Assessment

**Figure 2:**
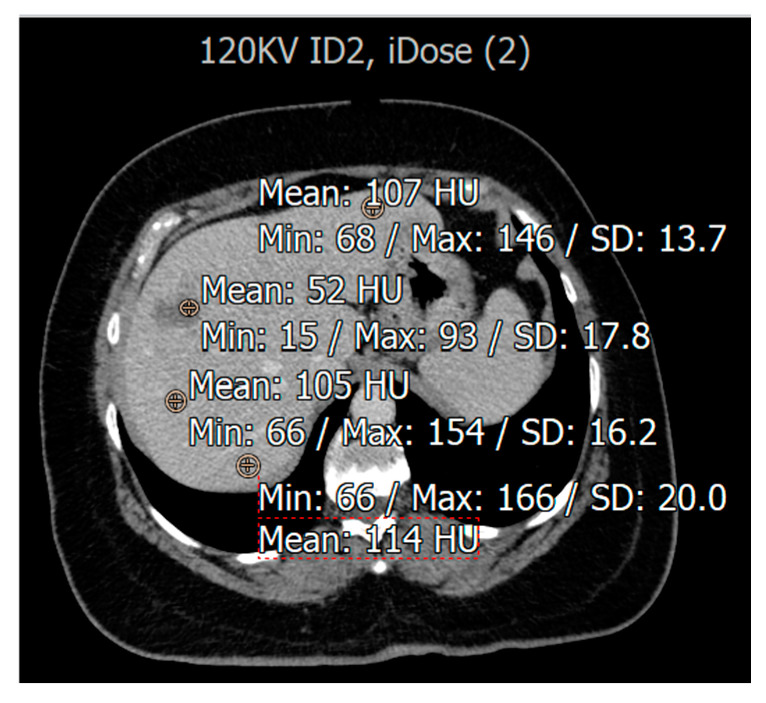
An illustration of HU measurement in region of interest (ROI) of liver CT images from a patient with liver hemangioma.

The study employed signal-to-noise ratio (SNR) and contrast-to-noise ratio (CNR) as evaluation metrics to assess the quality of CT images derived from eight different combinations. To determine the Hounsfield units (HUs), circular regions of interest (ROIs) consisting of 20 pixels were carefully drawn in three distinct normal locations within the liver CT images. These same ROIs were then replicated in the center of the lesion to measure the HU value for each area, as shown in Figure 2. Subsequently, the SNR was calculated through specific equations designed for this purpose [27], considering the variations in noise and signal strength in the images captured for analysis.

Normal SNR = average HU liver/SD liver

Abnormal SNR = HU lesion/SD liver

Normal CNR = HU liver − HU muscle/SD muscle

Abnormal CNR = HU lesion − HU muscle/SD muscle

**Figure 3:**
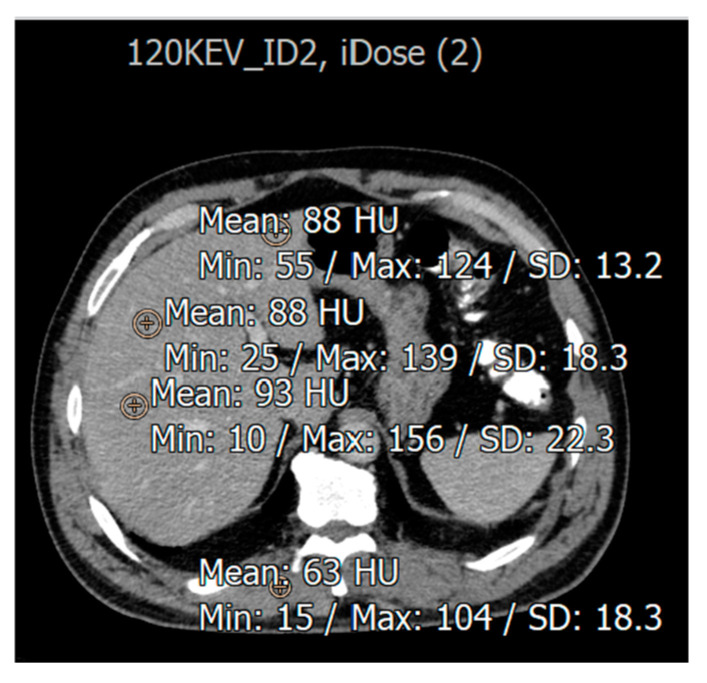
An illustration of HU measurement in region of interest (ROI) of liver CT images from a patient with fatty liver disease.

SNR and CNR were calculated for fatty liver disease (Figure 3) in the eight reconstructed CT images, using the following equations:

Abnormal SNR = HU lesion/SD liver

Abnormal CNR = HU lesion-HU muscle/SD muscle

In the data analysis phase, the statistical software SPSS (v 26, Chicago, IL, USA) was used to calculate the mean and standard deviations (SD). These calculations served as important metrics for evaluating liver CT images to distinguish between normal and abnormal cases. The SNR and CNR relating to metastasis lesions, hemangiomas, and fatty liver were also examined. The descriptive statistics encompassed an extensive comparison of eight combinations derived from three raters’ evaluations.

To thoroughly evaluate the radiation dose received from the abdomen and pelvis examination spectral CT scan, a comprehensive analysis was conducted by measuring the system’s CT dose index (CTDI) and dose length product (DLP) values. These values were then meticulously compared with the corresponding data available from multi-detector computed tomography (MDCT) scans acquired using a 64-slice detector of the same brand. All these intricate measurements were carried out using a body CT phantom designed to mimic a realistic human body structure, specifically, a PMMA cylinder phantom with a diameter of 32 cm for adult body scans.

The parameters used as standards for abdomen and pelvis CT examinations were similarly applied to both CT systems. For spectral CT, the typical parameters were 120 keV, 203 mAs, and a slice thickness of 3 mm. In contrast, multidetector CT usually employs 120 kV, 300 mAs, and a slice thickness of 3 mm. These parameters are essential for ensuring consistency and accuracy in examination procedures. The CTDIvol and DLP were the standard values used to assess the radiation dose from both systems.

The Pearson correlation coefficient (r) ranges were utilized to establish a framework for interpreting the level of agreement among the data. Values between 0.2 and 0.39 were classified as weak, 0.4 to 0.59 as moderate, and 0.6 to 0.79 as strong. Additionally, the percentage difference for the SNR (signal-to-noise ratio) data was carefully calculated to highlight variations. Statistical significance was assessed by analyzing *p*-values, using a significance level of 0.05. These systematic analyses and assessments provided valuable insights into the imaging data, ultimately enhancing our understanding of the study’s outcomes.

## 3. Results

The Pearson correlation and *p*-values for the various reconstruction techniques at different keV values are presented in Table 3. A positive relationship was observed as the tube voltage increased from 70 keV to 120 keV. Specifically, when comparing the IMR3 reconstruction technique at 120 keV with IMR3 at 70 keV, the correlation coefficient was 0.789. This indicates a strong positive correlation, and the result is statistically significant with a *p*-value of 0.000. In contrast, comparison of the iDOSE2 reconstruction technique at 120 keV with iDOSE2 at 70 keV exhibited the lowest correlation value at 0.291, suggesting a weak positive correlation, with a *p*-value of 0.045.

### 3.1. Imaging Assessment of Metastatic and Hemangioma Cases

**Figure 4:**
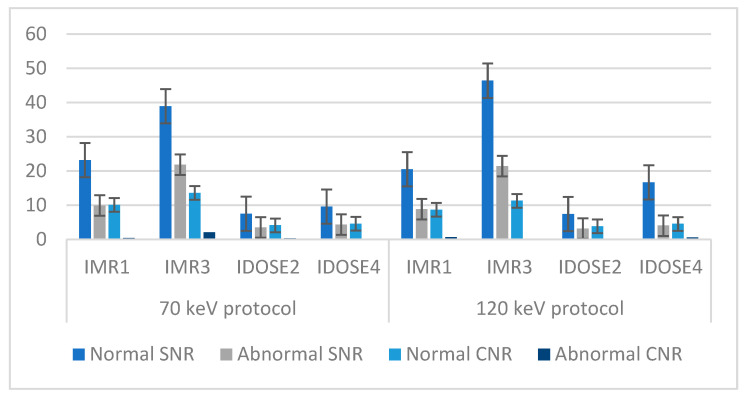
The mean SNR and CNR for various tube voltages and image reconstruction methods for patients with liver metastatis or hemangioma.

Based on the statistical analysis, it was found that the IMR3 reconstruction technique had almost the highest values for normal and abnormal SNR and normal CNR, as shown in Figure 4 and Table S1. The mean values for normal SNR using IMR3 with 70 keV and 120 keV were found to be the highest, at 38.92 and 46.4, respectively. The lowest value for normal SNR (7.56) was found using iDOSE 2 with 70 keV, and the normal SNR with 120 keV was found to be 7.42. The second parameter that was calculated was abnormal SNR; the IMR3 reconstruction combined with a low energy level resulted in 21.83, but with a high energy level, the value was found to be 21.47. Also, the lowest value for abnormal SNR using iDOSE 2 (70 keV) was 3.51; in contrast, iDOSE 2 (120 keV) had 3.21. The normal CNR using IMR3 with 70 keV was 13.63, but with 120 keV, the value was 11.32. The lowest CNR value was also found using iDOSE2. The abnormal CNR using IMR3 with 70 keV (2.11) was found to be higher than IMR1-70 (0.47), while in comparison with 120 keV energy, IMR1 showed a higher value (0.72) compared with IMR3 with the same energy level (0.23).

### 3.2. Evaluation for Patients with Fatty Liver Disease

**Figure 5:**
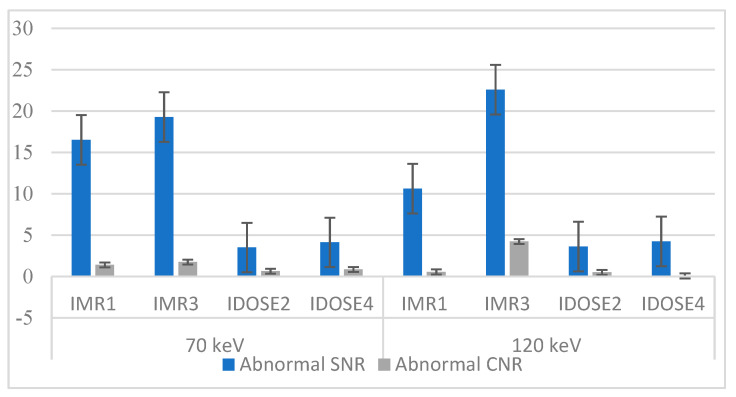
The mean values of SNR and CNR for various tube voltages and image reconstruction methods for patients with fatty liver.

Figure 5 and Table S2 display the mean signal-to-noise ratio (SNR) and contrast-to-noise ratio (CNR) for patients with fatty liver disease, using different reconstruction algorithms with 70 keV and 120 keV tube voltages. In the abnormal areas, the SNR achieved with the IMR3 reconstruction algorithm yielded the highest values at both energy levels. Conversely, the SNR for IDOSE2 at 70 keV was the lowest. Regarding abnormal CNR, the iDose4 algorithm with 120 keV resulted in the lowest value (0.07), while the highest value was observed with IMR3 with 120 keV, which was 4.22.

### 3.3. Image Quality Evaluation Based on Radiologist

**Figure 6:**
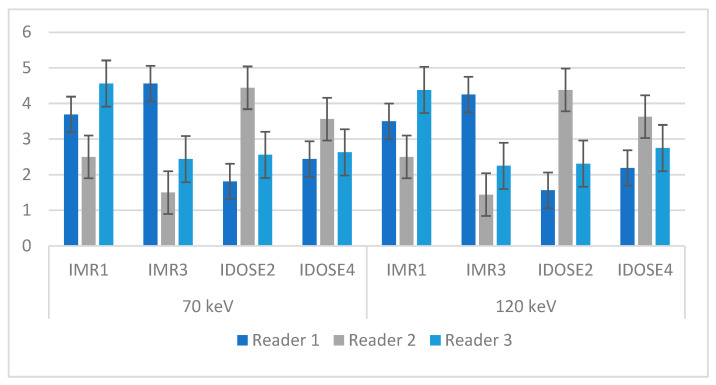
The mean and standard deviation for the reconstruction techniques with different keV selections, based on reader evaluation.

Figure 6 and Table S3 illustrate the mean output results obtained from three radiologists’ assessments based on eight reconstructed combinations. Based on the assessment, Reader 1 selected IMR3 with 70 keV as the best image in terms of visibility (4.56 ± 0.63) whereas iDOSE2 with 120 keV had the lowest image quality (1.56 ± 0.63). The second reader selected iDOSE2 with 70 keV as the best image quality (4.44 ± 0.62) and IMR3 with 120 keV as the worst image quality based on reader evaluation (1.44 ± 0.51).

The third reader chose the IMR1 and 70 keV as the best reconstruction method and energy value to diagnose the targeted pathologies (4.56 ± 0.51) while the IMR3–120 keV combination provided the worst image quality in terms of diagnostic visibility (2.25 ± 1.07). The average mean value and SD according to this evaluator’s opinion indicated that IMR1-70 keV provided the best visibility for the diagnosis of pathologies (3.58 with an SD of 0.986). On the other hand, the IMR3-120 keV resulted in the lowest image quality, with a mean value of 2.65 and an SD of 1.391.

The average mean value and SD obtained based the evaluators’ opinions showed that IMR1-70 keV provided the best visibility in the diagnosis of pathologies (3.58 with an SD of 0.986). Meanwhile, IMR3-120 keV produced the lowest image quality with a mean value of 2.65 and SD of 1.391 (Table 4).

The measured CTDIvol for SDCT and MDCT were 23.3 mGy and 32.2 mGy, respectively, while the DLP values for SDCT and MDCT were 628 mGy*cm and 775.2 mGy*cm, respectively. The percentage differences in CTDIvol and DLP between SDCT and MDCT were 32% and 20.9%, respectively.

## 4. Discussion

This study highlights the significant role of the new imaging device, Spectral CT, which greatly enhances diagnostic accuracy while reducing radiation exposure for patients. It is well established that minimizing image noise in CT scans relies on advanced imaging processing techniques that aid in reconstructing high-quality images. Additionally, improper adjustment of tube voltage can adversely affect image quality, resulting in increased noise levels and a lower signal-to-noise ratio. Improvements in CT imaging are crucial for radiologists, as they assist in accurately diagnosing liver diseases. This has a direct impact on treatment management and can help reduce mortality rates. Studies have reported that two million people die from liver disease annually, accounting for 4% of all deaths [28]. About 90% of all primary liver malignancies and over 9% of all newly diagnosed cases are hepatocellular carcinoma (HCC) [29].

The current findings reveal a notable trend; changing the tube voltage in SDCT while maintaining the same reconstruction algorithm leads to a significant enhancement in image quality. This correlation implies a clear relationship where higher kiloelectronvolts (keV) are associated with improved imaging performance. Furthermore, closer examination of the iterative model reconstruction (IMR) technique indicates that varying the level of reconstruction yielded higher Pearson correlation coefficients, ranging from moderate to strong positive correlations. This contrasts sharply with the iterative dose reduction (iDose) levels, which exhibited only weak positive correlations. Specifically, the Pearson correlation coefficients for IMR illustrate a strong upward trend in correlation with increased IMR levels. In comparison, the iDose levels showed notably lower coefficients. These findings align closely with the research conducted by Iyama et al. [30], which also assessed the Pearson correlation when comparing IMR with iDose and hybrid iterative reconstruction (HIR), in addition to FBP. That study revealed that IMR exhibited the highest correlation coefficient (r = 0.98), maintaining a significant advantage over other methods, followed by FBP (r = 0.41) and HIR (r = 0.33) [30]. These disparities in performance can be attributed to the underlying assumptions present in these CT systems. Previous reconstruction techniques, such as FBP, were based on simplified models, including linear X-ray beams, point source focal spot methods, and CT detector cells with uniform responses. In contrast, the IMR method employs precise system models to generate imaging data that more accurately align with the actual measured projection data [24]. Consequently, the IMR technique not only enhances image resolution but also improves the repeatability of interobserver assessments, while effectively reducing noise. These advantages are strongly corroborated by the results obtained, which further validate the superior efficacy of IMR in producing high-quality imaging. In a recent study, the image quality was significantly improved by 83% using the IMR method compared with iDose and FBP, while noise was reduced by 89% using the IMR method [31].

The data indicate that the SNR for abnormal liver tissues is consistently lower than that for healthy liver tissues, as referred in the comparative analysis of SNR between normal and abnormal hepatic conditions (Figure 1). This disparity is attributed to the inherent characteristics of liver lesions, which often exhibit hypoattenuation properties [32]. Further observations revealed that liver lesions classified as hypointense demonstrate an improved SNR when imaged at a lower energy level of 70 keV, compared with higher levels like 120 kV, across various reconstruction techniques. The quantitative SNR results showed that IMR3 had the highest SNR value, particularly when using 70 keV (21.83). In contrast, the SNR values at a higher energy level of 120 keV were slightly diminished when using the same reconstruction techniques, respectively. This confirms that an increase in SNR can be achieved by decreasing keV for abnormal liver lesions, which can be explained by the liver parenchyma’s heightened attenuation due to iodine uptake during the portal venous phase. This phenomenon, driven by an improved photoelectric effect, leads to increased attenuation values at lower tube voltages [33]. Supporting this result, other research has demonstrated statistically significant differences in image quality between datasets and a marked increase in SNR when comparing imaging at 70 kVp versus 120 kVp [34]. According to one study, the accuracy of low-voltage virtual monochromatic images (VMIs) had the greatest impact on image quality, indicating that spectral CT data can greatly enhance liver imaging, clinical workflow, and diagnostic precision [26]. In evaluating the contrast-to-noise ratio (CNR) values for both normal and abnormal liver tissues at various keV settings and reconstruction levels, it was observed that the CNR of normal liver tissues decreased as the keV increased. This indicates that as the energy level of the X-ray beam rises, the ability to distinguish between normal liver tissues and surrounding structures diminishes [35]. However, it was observed that the CNR improved as the level of reconstruction increased, regardless of the keV setting. This shows that higher reconstruction levels positively correlate with improved CNR, suggesting that utilizing advanced reconstruction techniques can enhance the visibility and distinction of abnormalities within the liver. These findings highlight the importance of considering both keV settings and reconstruction levels when evaluating the CNR in liver imaging. This aligns with a study that concluded that increasing the image reconstruction level is associated with roughly a 40% improvement in contrast-to-noise ratio (CNR). Additionally, this enhancement in CNR is further amplified by using lower tube voltage selections, which can increase CNR by up to 8%. Therefore, significantly enhanced image quality could be obtained by lowering the tube voltage while simultaneously increasing the level of iterative reconstruction, leading to notable noise reduction outcomes [36]. Interestingly, the results indicated a superior performance of the IMR algorithm compared to iDose, regardless of tube voltage variations, suggesting that IMR is more effective at managing noise levels than iDose. This assertion is supported by the findings reported by Kulkarni et al., who compared IMR with low-dose conventional CT and iDose 4 conventional CT specifically for HCC imaging. Utilizing low-dose multidetector computed tomography (MDCT) scans, they found that the IMR algorithm provided improved resolution and enhanced conspicuity of malignant lesions along with a significant reduction in image noise in comparison to the iDose 4 technique [24]. Another study compared iDose4 and IMR images with those obtained via conventional filtered back projection (FBP) to evaluate image quality in CT pulmonary angiography. Quantitative research revealed that IMR produced the highest CNR estimations, further proving its superiority over FBP and iDose4 in creating sharper images [37].

When assessing patients diagnosed with fatty liver disease, it is important to consider the impact of keV settings on the SNR across different imaging reconstruction algorithms. Notably, for the IMR1 technique, this study indicated a decrease in SNR as the keV increased from 70 to 120 keV. This behavior contrasts with other reconstruction methods such as IMR3, iDose2, and iDose4, where an increase in keV correlates with an improvement in SNR. The SNR tends to improve when higher levels of reconstruction are used across various techniques. This pattern is consistent with findings in the imaging of hemangiomas and metastatic lesions, where IMR reconstructions demonstrate better performance. Furthermore, it is crucial to consider how the choice of reconstruction technique affects image quality. Data analysis indicates that IMR3 stands out, offering higher image quality compared to its counterparts. The application of IMR3 in imaging for patients with fatty liver disease may enhance diagnostic accuracy and overall image clarity. By optimizing the keV settings and utilizing IMR3, radiologists can improve the quality of imaging, thereby aiding in both diagnosis and treatment planning for these patients.

Three experienced radiologists were consulted to provide their opinions on the best diagnostic approaches to liver disease, utilizing different reconstruction techniques and virtual tube voltages. The first radiologist preferred the IMR3 technique for diagnosing various liver pathologies, believing that it produced higher image quality compared with the iDose2 technique. This preference was based on the higher mean image quality achieved with IMR3. To further evaluate the performance of the different reconstruction methods, the radiologists also analyzed the effects of varying virtual tube voltage settings. Interestingly, the radiologist selected the images reconstructed at the lowest keV values, which consistently exhibited superior quality. This observation suggests that lower keV settings may contribute to improved image quality, potentially enhancing the accuracy of liver disease diagnoses.

The standard deviation (SD) of the measurements was taken into account in addition to the mean values when evaluating the quality of the images. The data points were found to be closely clustered around the mean, as evidenced by the remarkably small standard deviation. This tight clustering suggests that the image quality obtained from these reconstruction techniques was consistent. This consistency is vital as it enhances the diagnostic reliability of the imaging process, allowing more accurate and confident diagnosis of liver disease.

When comparing the results from the second radiologist, it was noticed that the quality ratings for images at 70 keV and 120 keV were closely aligned. Notably, the iDose 2 received the highest quality rating, while IMR3 was rated the lowest. In contrast, the findings from the third radiologist were significantly different, showing that images reconstructed at 70 keV were rated much higher than those reconstructed at 120 keV. Despite these differing opinions, it was concluded that IMR1 provided the highest image quality, while IMR3 was identified as resulting in the poorest.

The differences in the radiologists’ evaluations seem to have been influenced mainly by their varying levels of familiarity and experience with iDose technology compared with IMR. The second and third radiologists had significantly more experience with iDose, which probably led them to rate it more favorably than IMR. This variability in interpretation poses a significant challenge in radiological assessments. The outcomes of imaging techniques often depend on the subjective opinions of the observer, highlighting the potential for discrepancies in diagnostic conclusions [38]. If a radiologist becomes proficient with IMR images, they could potentially lower patients’ radiation exposure. Due to the noise reduction capabilities of this imaging method, the diagnostic potential of the produced images remains uncompromised. However, excessive noise reduction in low-dose acquisitions may result in image blurring, diminished resolution, and excessive smoothing of the image [39].

Comparison of interrater agreement showed that the IMR1 reconstruction at low doses (70 keV) provided the highest average scores for image quality. The data indicate that IMR1 reconstruction at reducing radiation doses, along with a minimal standard deviation (SD) of 0.98, resulted in superior image quality. Interestingly, the values for IMR1 at 120 keV were found to be very similar to those at 70 keV. In contrast, IMR3 consistently produced the lowest image quality scores, with a higher SD, which suggests greater variability and inconsistency in the results. This result may be attributed to the fact that iterative model reconstruction (IMR) is effective in enhancing the detectability of liver diseases by reducing image noise and improving the signal-to-noise ratio (SNR). However, increasing the level of IMR reconstruction can cause the CT images to appear “plastic” or give them a “blotchy” texture [39]. This might be the reason why IMR1 had the best image quality scores compared with the other reconstruction methods examined for assessing different liver diseases. From a different perspective, spectral CT provides a lower dose of radiation compared with conventional CT scans for standard abdomen and pelvis examinations. This advantage makes spectral CT a preferred choice for minimizing radiation exposure while delivering high-quality images. This is consistent with a previous study by Do et al., who found that it possible to reduce the radiation dose by 28% using spectral CT compared with conventional CT [40]. Overall, this evaluation involving the opinions and observations of three radiologists provides valuable insights into the performance and preferences of different reconstruction techniques for liver disease diagnosis. These findings can contribute to the improvement of clinical practice and the enhancement of patient care in the field of radiology.

This study encountered several limitations, including a relatively small sample size of patients and a retrospective design. Additionally, the radiologists’ experience with specific types of reconstructions may have influenced their decision-making regarding the most suitable reconstructions. Various factors can influence the image quality of spectral CT and its diagnostic capability for liver diseases, including the patient’s body mass index (BMI) and liver fat content. Therefore, further research could be conducted to examine how these factors interact with different reconstruction techniques and virtual tube voltage values.

## 5. Conclusions

In this study, significant enhancements in several image quality parameters were observed. Specifically, SNR values for both normal and abnormal liver tissues were markedly improved when utilizing SDCT with low radiation doses and a designated level of iterative reconstruction (IR). This indicates that SDCT not only maintains but enhances diagnostic effectiveness even under lower-dose conditions.

Additionally, the application of the IMR algorithm in the SDCT framework provided a notable boost in image quality and the conspicuity of liver lesions. The IMR algorithm was particularly effective in minimizing image noise, which is crucial for accurate assessment and diagnosis. Compared with other reconstruction techniques, the IMR algorithm demonstrated superior performance, suggesting that SDCT represents a promising advancement in liver imaging technologies. Overall, these findings highlight the efficacy of SDCT in delivering high-quality images of liver structures while ensuring patient safety through reduced radiation exposure based on virtual tube voltage, and this minimizes the requirement to repeat the CT scans while ensuring optimal detection of liver pathology.

## Figures and Tables

**Table 1:** Demographic characteristics of the participants (*N* = 47).

Variable	% (n)
Age Group	
18–29	6.3 (3)
30–39	19.1 (9)
40–59	38.3 (18)
60–75	36.1 (17)
Sex	
Male	59.6 (28)
Female	40.4 (19)
Type of liver pathology	
Liver metastasis	34 (16)
Hemangioma	34 (16)
Fatty liver	32 (15)

**Table 2:** The combination of reconstruction type and virtual tube voltage used in this study.

Reconstruction Techniques	iDose	IMR
Reconstructed image output	iDose2 70 keV	IMR1 70 keV
	iDose2 120 keV	IMR1 120 keV
	iDose4 70 keV	IMR3 70 keV
	iDose4 120 keV	IMR3 120 keV

**Table 3:** IMR and iDose reconstruction techniques with their correlation related to tube voltages.

Reconstruction Techniques	Pearson Correlation	*p*-Value
IMR1 120 keV vs. IMR1 70 keV	0.462 (**)	0.001
IMR3 120 keV vs. IMR3 70 keV	0.789 (**)	0.000
iDose2 120 keV vs. iDose2 70 kev	0.291 (*)	0.045
iDose4 120 keV vs. iDose4 70 keV	0.390 (**)	0.006

* weak postitve corelation. ** strong postive coreltion.

**Table 4:** The average mean value and standard deviation for all interrater evaluations.

Parameters	Mean	Std. Deviation
IMR1_70 kev	3.58	0.986
IMR3_70 kev	2.83	1.534
iDose2_70 kev	2.94	1.295
iDose4_70 kev	2.88	0.815
IMR1_120 kev	3.46	0.988
IMR3_120 kev	2.65	1.391
iDose2_120 kev	2.75	1.391
iDose4_120 kev	2.85	0.945

## Data Availability

Upon request, the main author will provide all related case data.

## Supplementary Materials

The following supporting information can be downloaded at https://www.mdpi.com/article/10.3390/diagnostics15081043/s1, Table S1: Descriptive statistics for various tube voltage and image reconstruction for patients with metastatic and hemangioma; Table S2: Descriptive statistics for various tube voltage and image reconstruction for patients with fatty liver; Table S3: The mean and standard deviation for the reconstruction techniques with different keV selections based on reader evaluation.

## Abbreviations

The following abbreviations are used in this manuscript:

SDCT	Spectral detector computed tomography
SNR	Signal-to-noise ratio
CNR	Contrast-to-noise ratio
IMR	Iterative model reconstruction
FBP	Filtered back projection
MDCT	Multidetector computed tomography
