# Supplementary material for: Assessment of Spectral Computed Tomography Image Quality and Detection of Lesions in the Liver Based on Image Reconstruction Algorithms and Virtual Tube Voltage
Source: Diagnostics (Basel). 2025 Apr 19;15(8):1043. doi: 10.3390/diagnostics15081043 (PMC12025537; doi:10.3390/diagnostics15081043)
Supplement: Supplementary file 1 [file diagnostics-15-01043-s001.zip › diagnostics-3563056-supplementary.pdf]

Table S1: Descriptive statistics for various tube voltage and image reconstruction for patients with metastatic and hemangioma

	70 keV protocol				120 keV protocol			
**Parameters**	**IMR1**	**IMR3**	**IDOSE2**	**IDOSE4**	**IMR1**	**IMR3**	**IDOSE2**	**IDOSE4**
**Normal SNR**	23.18±1.2	38.92±4.04	7.56±2.36	9.6±3.77	20.53±5.20	46.4±9.05	7.42±2.87	16.70±4.22
**Abnormal SNR**	9.93±5.45	21.83±9.5	3.51±2.02	4.36±2.61	8.87±5.64	21.47±6.83	3.21±2.23	4.07±2.72
**Normal CNR**	10.14±5.74	13.63±1.39	4.15±1.73	4.61±1.73	8.68±4.81	11.32±7.69	3.88±1.4	4.57±2.25
**Abnormal CNR**	0.47±6.15	2.11±7.86	0.32±1.34	0.29±1.77	0.72±4.51	0.23±7	0.0±2.03	0.57±1.91

Table S2: Descriptive statistics for various tube voltage and image reconstruction for patients with fatty liver

	70 keV protocol				120 keV protocol			
**Parameters**	**IMR1**	**IMR3**	**IDOSE2**	**IDOSE4**	**IMR1**	**IMR3**	**IDOSE2**	**IDOSE4**
**Abnormal SNR**	16.53±4.74	19.28±8.77	3.5±1.63	4.12±1.8	10.63±5.35	22.6±9.2	3.62±1.82	4.25±1.64
**Abnormal CNR**	1.40±3.66	1.75±8.98	0.63±1.42	0.85±1.88	0.55±3.06	4.22±10.89	0.5±1.28	0.07±1.41

Table S3: The mean and standard deviation for the reconstruction techniques with different keV selections based on reader evaluation

**Readers**	70 keV protocol				120 keV protocol			
	**IMR1**	**IMR3**	**IDOSE2**	**IDOSE4**	**IMR1**	**IMR3**	**IDOSE2**	**IDOSE4**
**Reader 1**	3.69±0.48	4.56±0.63	1.81±0.65	2.44±0.63	3.50±0.73	4.25±0.45	1.56±0.63	2.19±0.66
**Reader 2**	2.50±.52	1.50±0.52	4.44±0.63	3.56±0.51	2.50±0.52	1.44±0.51	4.38±0.72	3.63±0.50
**Reader 3**	4.56±0.51	2.44±1.21	2.56±0.73	2.63±0.81	4.38±0.62	2.25±1.07	2.31±0.79	2.75±1
